# Laboratory Parameters of Hemostasis, Adhesion Molecules, and Inflammation in Type 2 Diabetes Mellitus: Correlation with Glycemic Control

**DOI:** 10.3390/ijerph17010300

**Published:** 2020-01-01

**Authors:** Eleonora Palella, Rossella Cimino, Salvatore A. Pullano, Antonino S. Fiorillo, Elio Gulletta, Antonio Brunetti, Daniela P. Foti, Marta Greco

**Affiliations:** Department of Health Sciences, University “Magna Græcia” of Catanzaro, Viale Europa, 88100 Catanzaro, Italy; eleonora.palella@gmail.com (E.P.); rossellacimino@hotmail.it (R.C.); pullano@unicz.it (S.A.P.); nino@unicz.it (A.S.F.); gulletta@unicz.it (E.G.); brunetti@unicz.it (A.B.)

**Keywords:** type 2 diabetes mellitus, prothrombotic risk, hemostasis, inflammation, adhesion molecules

## Abstract

Background: Type 2 diabetes mellitus (T2DM) is characterized by a prothrombotic state, predisposing to vascular complications. Some related markers, linking thrombophilia to hemostasis and inflammation, however, have been poorly explored in relation to patients’ glycemia. We therefore investigated the association of laboratory hemostatic parameters, circulating adhesion molecules (ADMs), white blood cell (WBC) count, and neutrophil/lymphocyte ratio (NLR) with T2DM and glycemic control. Research design: In this study, 82 subjects, grouped into T2DM patients (*n* = 41) and healthy individuals (*n* = 41) were enrolled. To evaluate glycemic control, the T2DM cohort was expanded to 133 patients and sub-classified according to glycated hemoglobin (HbA1c) <7% and ≥ 7% (*n* = 58 and *n* = 75, respectively). We assessed glycemia, HbA1c, prothrombin time (PT), activated partial thromboplastin time (aPTT), fibrinogen, plasminogen activator inhibitor-1 (PAI-1), platelet and leukocyte parameters, vascular cell adhesion molecule 1 (VCAM-1), intercellular adhesion molecule 1 (ICAM-1), and selectins (E-, P-, L-). Results: PT % activity, PAI-1, VCAM-1, WBC, and neutrophil counts were significantly higher in T2DM patients than in healthy subjects. Poor glycemic control (HbA1c ≥ 7%) was correlated with increased PT activity (*p* = 0.015), and higher levels of E-selectin (*p* = 0.009), P-selectin (*p* = 0.012), and NLR (*p* = 0.019). Conclusions: Both T2DM and poor glycemic control affect some parameters of hemostasis, inflammation, and adhesion molecules. Further studies are needed to establish their clinical utility as adjuvant markers for cardio-vascular risk in T2DM patients.

## 1. Introduction

Type 2 diabetes mellitus (T2DM) is characterized by chronic hyperglycemia, and by a 2–4-fold increased risk of atherosclerotic cardiovascular disease (CVD), leading, in nearly 80% of patients, to a fatal thrombotic event [1,2]. In line with these epidemiological data, several studies have shown that T2DM predisposes to a multifactorial prothrombotic state, in which endothelial dysfunction, platelet hyper-reactivity, alterations of the coagulation cascade, impaired fibrinolysis, as well as chronic, low-grade inflammation, are known to play a major role [3,4,5,6].

Vascular endothelial surface regulates the balance between coagulation and fibrinolysis, the adhesion and extravasation of leucocytes, and the inflammatory activity within the vessel wall. It also releases different molecules and mediators involved in vascular homeostasis, in platelet activation, in the coagulation cascade [7,8], as well as in the fibrinolytic system, such as the tissue plasminogen activator (t-PA), and the plasminogen activator inhibitor-1 (PAI-1) [9]. Dysfunction of the vascular endothelium, as well as alterations in its permeability, are involved in the formation and progression of the atherosclerotic plaque, and in the pathogenesis of angiopathic complications in T2DM patients, predisposing to adverse outcomes [10]. In addition, in T2DM, the coagulation cascade is substantially activated. In particular, the tissue factor (TF), as well as factor VII (FVII), factor VIII (FVIII), von Willebrand factor (vWF), and fibrinogen, have been reported to be increased [2,5]. While a derangement of the anti-coagulation factors has been mainly associated with venous thrombosis, impaired fibrinolysis in T2DM patients is strongly involved in the genesis of vascular arterial complications. The inhibition of fibrinolytic activity in diabetic patients is mainly due to an increase in PAI-1 levels [5,9], a condition that represents a risk factor for thrombosis and atherosclerosis [4,11]. Meta-inflammation, mainly sustained by obesity [12], also plays a role in predisposing T2DM patients to thrombophilia. This involves macrophages located in the adipose tissue, liver, and gastro-intestinal tract through the release of several cytokines that promote, contextually, insulin resistance, endothelial dysfunction, coagulation, and PAI-1 biosynthesis [13,14].

It is well known that T2DM patients are recommended to maintain glycemic standards based on epidemiological data in order to prevent, or at least, delay the onset and progression of vascular complications [15,16]. Today, despite the need to identify prognostic markers to improve T2DM patients’ outcome, few and controversial studies show the correlation between glycemic control and laboratory biomarkers linked to the susceptibility to thrombosis in diabetic patients.

The aim of this study is focused on the investigation, through commonly used laboratory parameters, of the hemostatic process, the endothelial dysfunction, and inflammation in T2DM patients and their association with the degree of glycemic control, as evidenced by glycated hemoglobin (HbA1c) levels.

## 2. Materials and Methods

### 2.1. Study Design and Patients Enrolled

In this case-control study, 82 subjects, grouped into T2DM patients (*n* = 41) and healthy controls (*n* = 41) were initially enrolled. Healthy volunteers were consecutively recruited among the blood donors attending the provincial center “AVIS” of Catanzaro. Eligibility of controls was assessed after a brief medical interview aimed at excluding glycemic abnormalities, previous thrombotic events, chronic and acute diseases, and pharmacological treatments. Inclusion criteria were glycemia < 100 mg/dL, blood counts, renal, and hepatic function tests within the reference range in the routine laboratory analyses preceding blood donation. Patients with T2DM were recruited by a brief medical interview at the blood draw center of the Azienda Ospedaliero-Universitaria “Mater Domini” of Catanzaro, Later, in a second step of the study, to further investigate the role of glycemic control, the T2DM cohort was widened to 133 patients using the same recruitment procedures. Diabetic patients were diagnosed according to the American Diabetes Association (ADA) criteria. Patients with T2DM were included if under diet or treated with anti-hyperglycemic oral therapy, and if they reported diabetes duration < 10 years, no history of diabetic vascular complications or previous thrombotic events, such as myocardial infarction or ischemic stroke. The exclusion criteria from the study were: smoking, caffeine abuse, any pharmacological treatment such as heparin, warfarin, insulin, nitroglycerin, beta blockers, cortisone, and anti-depressants, intercurrent diseases during enrollment, such as inflammatory disease, severe obesity and clinical/laboratory evidence of chronic and debilitating diseases (e.g., cancer, cardiovascular disease, renal failure, liver dysfunction, and pulmonary disease). Patients and controls with acute conditions immediately before or after recruitment were excluded from the study.

Informed consent was obtained for each subject. The study protocol was designed according to the principles of the Declaration of Helsinki and approved by the local Ethical Committee (n. 2013/7, Comitato Etico, Azienda Ospedaliero-Universitaria “Mater Domini”, Catanzaro, Italy).

### 2.2. Laboratory Determinations

Blood samples were collected after 12–14 h overnight fasting and worked-out for routine analysis or for storage at −80 °C. Fasting glycemia, HbA1c, prothrombin time (PT); activated partial thromboplastin time (aPTT); fibrinogen, PAI-1; blood count analysis (white blood cells (WBCs) and differential counts, platelet parameters—platelet counts (PLT) and mean platelet volume (MPV)) were routinely analysed; adhesion molecules (ADMs) such as E-selectin, P-selectin, L-selectin, intercellular adhesion molecule-1 (ICAM-1) and vascular cell adhesion molecule-1 (VCAM-1) were evaluated after thawing −80 °C stored samples. Blood count analysis was performed using ADVIA 2120i (Siemens Healthcare Diagnostics, Marburg, Germany). Hemostatic parameters were determined on BCS XP (Siemens, Healthcare Diagnostics, Marburg, Germany), fasting glycemia was performed on Cobas 6000 (Roche Diagnostics, Risch-Rotkreuz, Switzerland). HbA1c was evaluated by high–performance liquid chromatography using the HA-8160™ HbA1c analyser (A. Menarini Diagnostics, Firenze, Italy). Circulating levels of ADMs were simultaneously measured by a multiplex testing using the “Randox Adhesion Molecules Array kit” and the Randox Evidence Investigator analyser (Randox Laboratories, Crumlin, UK). This assay is based on a two-site chemiluminescent sandwich immunoassay [17]. All the above-mentioned tests were carried out according to the manufacturer’s instructions. Quality controls were daily assessed for all the determinations, and results have met the precision targets indicated by the manufacturers.

### 2.3. Statistical Analysis

All the parameters analysed in the study were considered as continuous variables and expressed as average ± standard deviation (SD) after verification of their normal distribution by Kolmogorov–Smirnov test. The analysis was performed using the SPSS software version 20.0 (SPSS Inc., Chicago, IL, USA) and a *p* value < 0.05 was considered statistically significant. The independent Student's *t*-Test was used to evaluate the intergroup differences between any laboratory parameters. Pearson's correlation test and regression analysis were performed to evaluate the correlation between clinical and laboratory parameters.

## 3. Results

### 3.1. Comparison between Patients with T2DM and Controls

The clinical and biochemical characteristics of the population involved in the first phase of the study, which included a numerically identical group of T2DM patients vs. age-matched healthy controls, are summarized in Table 1.

Besides the expected differences between the metabolic parameters, T2DM patients vs. healthy controls showed significant differences in relation to the hemostatic parameters, i.e., PT % activity and PAI-1, supporting the concept of a hypercoagulative, and hypofibrinolytic status in T2DM (Table 1). All the other examined hemostatic indexes, including fibrinogen, PLT and MPV, show a trend towards a prothrombotic phenotype in T2DM, which did not reach significance probably due to the small sample size (Table 1). As cell ADMs are implicated in many processes, including inflammation, endothelial dysfunction and thrombosis, we determined the soluble fractions of some of these proteins in the serum of our study population. Among ADMs, a significant difference was observed for VCAM–1, whose level was increased in diabetic patients compared to healthy controls (Table 1). No significant differences were observed for ICAM-1, E-, P-, and L- selectins, although, in some of them, there was an increasing trend. Also, with respect to the chronic low-grade inflammation associated with T2DM, we analysed WBC. In our cohort, a significant increase in total WBC and absolute neutrophil counts was observed in T2DM patients compared to healthy controls (Table 1). Neutrophil/lymphocyte ratio (NLR), instead, was close to, but did not reach a significant difference, probably due to the small sample size.

### 3.2. Comparison between Patients with T2DM in Relation to the Degree of Glycemic Control

In a second step of the study, a correlation of the same parameters with the degree of glycemic control (HbA1c < 7% = good glycemic control; ≥ 7% = poor glycemic control) was investigated in an extended cohort of T2DM patients, matched for age. The characteristics of the extended cohort and the parameters measured are shown in Table 2.

Our results showed that, among the hemostatic parameters, PT% activity was significantly higher in T2DM patients with a worse glycemic control, while PAI-1 displayed an increasing trend. In relation to ADMs, in diabetic patients with HbA1c ≥ 7%, E-selectin and P-selectin were significantly increased, indicating that these selectins are presumably affected by different blood glucose levels. Furthermore, in relation to WBC, patients with poor glycemic control showed a higher NLR than patients with a good glycemic control.

### 3.3. Correlation Analyses between Glycemic Control and other Parameters

**Figure 1:**
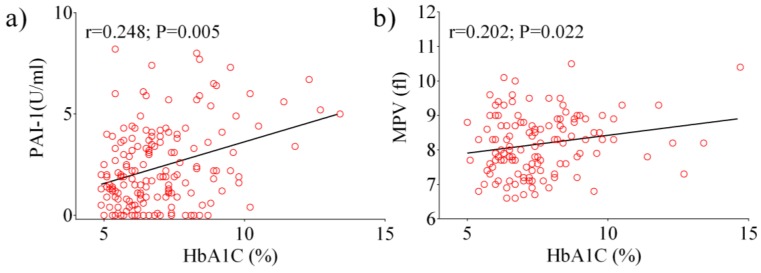
Pearson analysis showing the correlation between the laboratory parameters examined in the total diabetic population: HbA1c vs. PAI-1 (**a**), HbA1c vs. MPV (**b**).

**Figure 2:**
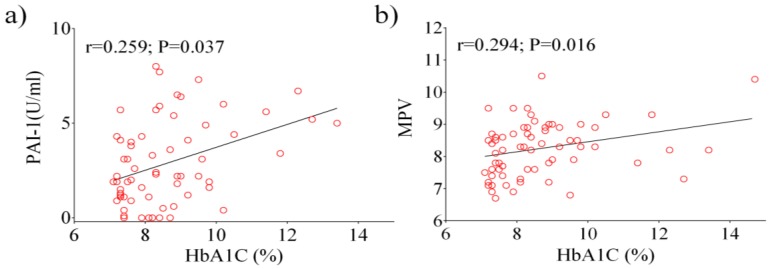
Pearson analysis showing the correlation between the laboratory parameters examined in patients with HbA1c ≥ 7%: HbA1c vs. PAI-1 (**a**), HbA1c vs. MPV (**b**).

Next, we analyzed data from the total diabetic population using a Pearson’s correlation test. A positive correlation between HbA1c, PAI-1 (*p* = 0.005), and MPV (*p* = 0.022) were observed (Figure 1a,b). Other significant correlations were observed between fasting glucose, PAI-1 (*p* < 0.0001), E-selectin (*p* = 0.029), ICAM-1 (*p* = 0.009), and P-selectin (*p* = 0.044). Following the analysis of patients with HbA1c ≥ 7%, a positive correlation was found between HbA1c, PAI-1 (*p* = 0.037) and MPV (*p* = 0.016) (Figure 2a,b), as well as between fasting glucose and E-selectin (*p* = 0.013).

## 4. Discussion

T2DM patients are known to be more susceptible than non-diabetic patients to macrovascular complications and thrombotic events [2,18]. Atherosclerotic CVD and T2DM have been reported to share a “common soil” [19], so that genetic, epigenetic, and environmental factors concur in their onset and progression [20,21]. Nevertheless, it has been highlighted that the increased prevalence of traditional risk factors for atherosclerosis in T2DM, such as dyslipidemia and hypertension, explains only half of the susceptibility of these patients to CVD [22], thereby additional elements, such as adiposity, should be taken into account in the pathogenesis of CVD in T2DM. In T2DM, CVD risk is, at least in part, due to a prothrombotic state as a consequence of several pathophysiological changes, including endothelial dysfunction, alterations in the hemostatic process (platelet hyper-reactivity, increased synthesis of coagulation factors, reduced fibrinolysis), as well as inflammation [1,2,5,14]. In this context, both hyperglycemia and/or insulin resistance may play an important pathogenetic role [5,23,24].

In this work, we investigated several laboratory parameters potentially linked to the thrombophilic status of T2DM, and analysed their association with the degree of glycemic control, as determined by HbA1c levels, in a homogeneous cohort of patients attending our campus. We evaluated PT and aPTT—two assays often used in conjunction—to test, respectively, the extrinsic and the common pathway of coagulation, and the intrinsic and common pathway of coagulation. In addition, we tested fibrinogen, the circulating precursor of fibrin, as well as PLT and MPV, an index of platelet activity and a risk factor for CVD [25]. Also, we measured PAI-1, the main inhibitor of fibrinolysis.

Among the analysed hemostatic parameters, PT% activity was significantly increased both in T2DM patients vs. controls, and in diabetic patients with poor vs. good glycemic profile expressed in terms of HbA1c (HbA1c < 7% vs. ≥ 7%), suggesting a hypercoagulability state in T2DM, that is mainly affected by high mean glucose levels. This result is coherent with previous reports indicating a more prominent synthesis of TF and FVII in diabetes [2,5].

A statistically significant increase in PAI-1 was observed in T2DM vs. control group, but not when diabetic patients with HbA1c < or ≥ 7% were compared. A weak positive correlation between PAI-1 and HbA1c, however, emerged from the Pearson’s test, besides a slight positive correlation between MPV and HbA1c.

Several studies have described the association of high concentrations of PAI-1 with chronic complications of diabetes, in particular retinopathy, nephropathy, and coronary heart disease [26,27], as well as with a poor prognosis for myocardial infarction in the acute phase [28]. Also, a link between PAI-1 and atherothrombosis via obesity and insulin resistance has been definitely assessed, being visceral fat a major regulator of PAI-1 [29,30]. As we observed an association of PAI-1 with T2DM, but not with the degree of glycemic control, our data are coherent with previous findings and with the concept that, in T2DM, the increase in PAI-1 levels impairs fibrinolysis mainly via insulin resistance.

ADMs play a crucial role in maintaining the balance of pro-inflammatory and procoagulant state by the adhesion processes of platelets and leukocytes at the endothelial surface and in the leukocyte transmigration through the vascular wall. Moreover, abnormalities in ADM expression play a central role in the development of endothelial dysfunction and in the different steps of atherogenesis [31,32,33]. In the present study, a significant increase in VCAM-1, an adhesion molecule known to be involved in the development of atherosclerosis [34], was observed in type 2 diabetic patients vs. controls, although no differences were seen in relation to glycemic control. E- and P- selectins, instead, were not associated with the disease per se, but with the levels of HbA1c.

Our findings are in part consistent with previous data. In a previous report by Blüher [35], E-selectin, ICAM-1 and VCAM-1 were all positively correlated with T2DM, if compared to euglycemic and impaired glucose tolerant patients. In a more recent work, Ruszkowska-Ciastek et al. [36] observed an increase in the blood concentrations of E-selectin and VCAM-1, and a decrease in ICAM-1 in T2DM patients with HbA1c ≥ 7.5% compared to patients with HbA1c < 6.5%. In both the above-mentioned reports, differences exist with our study in the experimental design and in the laboratory testing methodologies, as ADMs were measured in both cases by immune-enzymatic assays (E.L.I.S.A). Discrepancies between our results and data from other groups may also be due to differences in ethnicity, inclusion criteria and clinical characteristics of the study population, biological matrices to be tested, as well as laboratory methodologies.

Concerning P-selectin, an ADM involved in the initial phase of the atherosclerotic plaque formation, and a predictor of myocardial infarction [37,38], our findings are consistent with the data reported by the Multi-Ethnic Study of Atherosclerosis, which describes an association between P-selectin and HbA1c in all the ethnic groups examined [39]. Furthermore, a recent paper identified P-selectin and VCAM-1, as well as interleukin 18, as markers pathogenetically linked to, and predictive of diabetic nephropathy [40].

Total, differential number of WBC, and NLR have been investigated to evaluate the inflammatory state. It is known that WBC count is a valid marker of inflammation, that may be associated with the alteration of glucose metabolism, insulin resistance and T2DM [41,42]. Interestingly, elevated NLR is associated with, and represents a reliable predictive marker of insulin resistance [43], of the degree of glucose intolerance [44], as well as a risk factor for CVD [45]. Our results showed a statistically significant increase in WBC number, and neutrophils in patients with T2DM compared to healthy controls, with a higher NLR in patients with higher HbA1c levels. These data confirm the idea that the leukocytes represent a useful support in the evaluation of the inflammatory state in diabetic patients, and that, in agreement with the literature, NLR represents an adjuvant prognostic marker in T2DM able to predict vascular complications.

## 5. Conclusions

Alterations in the hemostasis and in the systemic inflammatory process underpinning T2DM may increase the risk of cardiovascular events in diabetic patients. Therefore, the identification of early predictive markers may help reduce diabetes–related vascular complications. Our results suggest that PT% activity, E-selectin, P-selectin, PAI-1, and NLR are significantly correlated with glycemic control and could be used as adjuvant prognostic markers for vascular complications in type 2 diabetic patients. Further studies are needed to investigate whether the combination of these conventional, low-cost and easy-to-use laboratory parameters may be helpful in clinical practice.

## Figures and Tables

**Table 1:** General characteristics and laboratory parameters of the study population.

	Control	T2DM	*p*
*N*	41 (13♀–28♂)	41 (22♀–19♂)	
Age (yrs)	51 ± 7.55	54 ± 5.10	0.95
Fasting glucose (mg/dL)	89 ± 4.86	161 ± 47.01	**<0.0001**
HbA1c (%)	5.4 ± 0.2	7.3 ± 1.5	**<0.0001**
**Hemostasis and platelet parameters**			
PT (%)	98.22 ± 8.54	105.49 ± 8.35	**<0.0001**
aPTT (s)	27.02 ± 2.56	26.76 ± 7.28	0.824
Fibrinogen (mg/dL)	297.51 ± 65.04	312.79 ± 74.62	0.345
PAI-1 (IU/mL)	2.02 ± 2.20	3 ± 1.7	**0.040**
PLT (10^3 /μL)	238.37 ± 67.18	247.49 ± 65.56	0.536
MPV (fl)	7.88 ± 0.69	8.12 ± 0.83	0.232
**Adhesion molecules**			
VCAM–1 (ng/mL)	290 ± 193.93	384 ± 191.36	**0.040**
ICAM–1 (ng/mL)	245 ± 116.69	282 ± 142.31	0.232
E-selectin (ng/mL)	13 ± 11.62	15 ± 8.45	0.381
P-selectin(ng/mL)	65 ± 45.49	65 ± 33.21	0.960
L-selectin (ng/mL)	839 ± 340.13	963 ± 415.97	0.206
**WBC and differential count**			
WBC (10^3/μL)	6.4 ± 1.4	7.6 ± 2.4	**0.009**
Neutrophils (10^3/μL)	3.6 ± 0.9	4.4 ± 1.7	**0.008**
Lymphocytes (10^3/μL))	2.1 ± 0.4	2.4 ± 0.9	0.082
NLR	1.7 ± 0.4	2.0 ± 0.8	0.067

Data are expressed as mean values ± standard deviation (SD). The inter-group variability is determined by the independent Student’s *t*-Test; significance level of *p* value is < 0.05. HbA1c = glycated hemoglobin; PT = prothrombin time; aPTT = activated partial thromboplastin time; PAI-1 = plasminogen activator inhibitor-1; PLT = platelets; MPV = mean platelet volume; vascular cell adhesion molecule-1 (VCAM-1); intercellular adhesion molecule-1 (ICAM-1); white blood cell (WBC) count; NLR = neutrophils/lymphocytes ratio.

**Table 2:** General characteristics and laboratory parameters of the diabetic patients involved in the second phase of the study.

	HbA1c < 7%	HbA1c ≥ 7%	*p*
*N*	58 (30♀–28♂)	75 (41♀–34♂)	
Age (yrs)	64.63 ± 10.9	66.43 ± 10.4	0.340
Fasting glucose (mg/dL)	126.89 ± 20.9	181.04 ± 66.4	**<0.0001**
HbA1c (%)	6.2 ± 0.4	8.5 ± 1.6	**<0.0001**
**Hemostasis and platelet parameters**			
PT (%)	99.37 ± 13.5	104.10 ± 8.2	**0.015**
aPTT (s)	26.51 ± 3.2	25.89 ± 5.8	0.474
Fibrinogen (mg/dL)	333.19 ± 97.2	338.75 ± 86.7	0.739
PAI–1 (IU/mL)	2.23 ± 1.8	2.79 ± 2.1	0.125
PLT (10^3/μL)	232.43 ± 70.6	235.76 ± 56.6	0.764
MPV (fl)	8.12 ± 0.9	8.17 ± 0.9	0.750
**Adhesion molecules**			
VCAM-1 (ng/mL)	458.07 ± 371.0	451.99 ± 236.4	0.909
ICAM-1 (ng/mL)	277.9 ± 140.9	274.11 ± 133.6	0.877
E–selectin (ng/mL)	12.42 ± 8.4	18.36 ± 15.2	**0.009**
P–selectin (ng/mL)	52.64 ± 34.1	70.08 ± 42.4	**0.012**
L–selectin (ng/mL)	921.54 ± 361.9	1008.97 ± 463.80	0.282
**WBC and differential count**			
WBC (10^3/μL)	7.48 ± 2.1	7.61 ± 2.2	0.746
Neutrophils (10^3/μL)	4.20 ± 1.5	4.60 ± 1.8	0.173
Lymphocytes (10^3/μL)	2.45 ± 1.1	2.17 ± 0.7	0.082
NLR	1.90 ± 0.8	2.28 ± 0.97	**0.019**

Data are expressed as mean values ± standard deviation (SD). The inter–group variability is determined by the independent Student’s *t*-Test; significance level of *p* value is < 0.05; HbA1c = glycated hemoglobin; PT = prothrombin time; aPTT = activated partial thromboplastin time; PAI-1=plasminogen activator inhibitor-1; PLT = platelets; MPV = mean platelet volume; vascular cell adhesion molecule-1 (VCAM–1); intercellular adhesion molecule-1 (ICAM-1); white blood cell (WBC) count; NLR = neutrophils/lymphocytes ratio.

## References

[1] Fox C.S., Coady S., Sorlie P.D., Levy D., Meigs J.B., D’Agostino R.B., Wilson P., Savage P.J. (2004). Trends in cardiovascular complications of diabetes. JAMA.

[2] Carr M.E. (2001). Diabetes mellitus: A hypercoagulable state. J. Diabetes Complicat..

[3] Dunn E.J., Ariëns R.A., Grant P.J. (2005). The influence of type 2 diabetes on fibrin structure and function. Diabetologia.

[4] Paneni F., Costantino S., Cosentino F. (2014). Insulin resistance, diabetes, and cardiovascular risk. Curr. Atheroscler. Rep..

[5] Grant P.J. (2007). Diabetes mellitus as a prothrombotic condition. J. Intern. Med..

[6] Shoelson S.E., Lee J., Goldfine A.B. (2006). Inflammation and insulin resistance. J. Clin. Investig..

[7] Rask-Madsen C., King G.L. (2007). Mechanisms of disease: Endothelial dysfunction in insulin resistance and diabetes. Nat. Clin. Pract. Endocrinol. Metab..

[8] Jansson PA. (2007). Endothelial dysfunction in insulin resistance and type 2 diabetes. J. Intern. Med..

[9] Auwerx J., Bouillon R., Collen D., Geboers J. (1988). Tissue-type plasminogen activator antigen and plasminogen activator inhibitor in diabetes mellitus. Arteriosclerosis.

[10] Simionescu M. (2007). Implications of early structural-functional changes in the endothelium for vascular disease. Arterioscler. Thromb. Vasc. Biol..

[11] Vaughan D.E. (2005). PAI-1 and atherothrombosis. J. Thromb. Haemost..

[12] Caroleo M., Carbone E.A., Greco M., Corigliano D.M., Arcidiacono B., Fazia G., Rania M., Aloi M., Gallelli L., Segura-Garcia C. (2019). Brain-Behavior-Immune Interaction: Serum Cytokines and Growth Factors in Patients with Eating Disorders at Extremes of the Body Mass Index (BMI) Spectrum. Nutrients.

[13] Dali-Youcef N., Mecili M., Ricci R., Andrés E. (2013). Metabolic inflammation: Connecting obesity and insulin resistance. Ann. Med..

[14] Ziegler D. (2005). Type 2 diabetes as an inflammatory cardiovascular disorder. Curr. Mol. Med..

[15] Stolar M. (2010). Glycemic control and complications in type 2 diabetes mellitus. Am. J. Med..

[16] American Diabetes Association (2019). Glycemic targets: Standards of Medical Care in Diabetes-2019. Diabetes Care.

[17] Accattato F., Greco M., Pullano S.A., Caré I., Fiorillo A.S., Pujia A., Montalcini T., Foti D.P., Brunetti A., Gulletta E. (2017). Effects of acute physical exercise on oxidative stress and inflammatory status in young, sedentary obese subjects. PLoS ONE.

[18] Sarwar N., Gao P., Seshasai S.R., Gobin R., Kaptoge S., Di Angelantonio E., Ingelsson E., Lawlor D.A., Selvin E., Stampfer M. (2010). Diabetes mellitus fasting blood glucose concentration and risk of vascular disease: A collaborative meta-analysis of 102 prospective studies. Lancet.

[19] Stern M.P. (1995). Diabetes and cardiovascular disease: The “common soil” hypothesis. Diabetes.

[20] Brunetti A., Chiefari E., Foti D. (2014). Recent advances in the molecular genetics of type 2 diabetes mellitus. World J. Diabetes.

[21] De Rosa S., Arcidiacono B., Chiefari E., Brunetti A., Indolfi C., Foti D.P. (2018). Type 2 diabetes mellitus and cardiovascular disease: Genetic and epigenetic links. Front. Endocrinol (Lausanne).

[22] Kannel W.B., McGee D.L. (1979). Diabetes and cardiovascular disease. The Framingham study. JAMA.

[23] Bansilal S., Farkouh M.E., Fuster V. (2007). Role of insulin resistance and hyperglycemia in the development of atherosclerosis. Am. J. Cardiol..

[24] Stegenga M.E., van der Crabben S.N., Levi M., de Vos A.F., Tanck M.W., Sauerwein H.P., van der Poll T. (2006). Hyperglycemia stimulates coagulation, whereas hyperinsulinemia impairs fibrinolysis in healthy humans. Diabetes.

[25] Pafili K., Penlioglou T., Mikhailidis D.P., Papanas N. (2019). Mean platelet volume and coronary artery disease. Curr. Opin. Cardiol..

[26] Azad N., Agrawal L., Emanuele N.V., Klein R., Bahn G.D., McCarren M., Reaven P., Hayward R., Duckworth W., VADT Study Group (2014). Association of PAI-1 and fibrinogen with diabetic retinopathy in the Veterans Affairs Diabetes Trial (VADT). Diabetes Care.

[27] Nicholas S.B., Aguiniga E., Ren Y., Kim J., Wong J., Govindarajan N., Noda N., Wang W., Kawano Y., Collis A. (2005). Plasminogen activator inhibitor-1 deficiency retards diabetic nephropathy. Kidney Int..

[28] Alessi M.C., Juhan-Vague I. (2004). Contribution of PAI-1 to cardiovascular pathology. Arch. Mal. Coeur Vaiss..

[29] Meigs J.B., Mittleman M.A., Nathan D.M., Tofler G.H., Singer D.E., Murphy-Sheehy P.M., Lipinska I., D’Agostino R.B., Wilson P.W. (2000). Hyperinsulinemia. hyperglycemia. and impaired hemostasis: The Framingham Offspring Study. JAMA.

[30] Juhan-Vague I., Alessi M.C., Mavri A., Morange P.E. (2003). Plasminogen activator inhibitor-1. inflammation. obesity. insulin resistance and vascular risk. J. Thromb. Haemost..

[31] Wang C.C.L., Hess C.N., Hiatt W.R., Goldfine A.B. (2016). Atherosclerotic Cardiovascular Disease and Heart Failure in Type 2 Diabetes—Mechanisms, Management, and Clinical Considerations. Circulation.

[32] Lusis A.J. (2000). Atherosclerosis. Nature.

[33] Oishi Y., Wakatsuki T., Nishikado A., Oki T., Ito S. (2000). Circulating adhesion molecules and severity of coronary atherosclerosis. Coron. Artery Dis..

[34] Wautier J.L., Wautier M.P. (2001). Blood cells and vascular cell interactions in diabetes. Clin. Hemorheol. Microcirc..

[35] Blüher M., Unger R., Rassoul F., Richter V., Paschke R. (2002). Relation between glycaemic control, hyperinsulinaemia and plasma concentrations of soluble adhesion molecules in patients with impaired glucose tolerance or Type II diabetes. Diabetologia.

[36] Ruszkowska-Ciastek B., Sokup A., Wernik T., Ruprecht Z., Góralczyk B., Góralczyk K., Gadomska D., Rość D. (2015). Effect of uncontrolled hyperglycemia on levels of adhesion molecules in patients with diabetes mellitus type 2. J. Zhejiang Univ. Sci. B.

[37] Ridker P.M., Buring J.E., Rifai N. (2001). Soluble P-selectin and the risk of future cardiovascular events. Circulation.

[38] Zalewski G., Ciccarone E., Di Castelnuovo A., Zito F., Capani F., de Gaetano G., Donati M.B., Iacoviello L., GENDIABE investigators. (2006). P–selectin gene genotypes or haplotypes and cardiovascular complications in type 2 diabetes mellitus. Nutr. Metab. Cardiovasc. Dis..

[39] Bielinski S.J., Berardi C., Decker P.A., Kirsch P.S., Larson N.B., Pankow J.S., Sale M., de Andrade M., Sicotte H., Tang W. (2015). P-selectin and subclinical and clinical atherosclerosis: The Multi-Ethnic Study of Atherosclerosis (MESA). Atherosclerosis.

[40] Al-Rubeaan K., Nawaz S.S., Youssef A.M., Al Ghonaim S.K., Siddiqui K. (2019). IL-18, VCAM-1 and P-selectin as early biomarkers in mormoalbuminuric Type 2 diabetes patients. Biomark. Med..

[41] Twig G., Afek A., Shamiss A., Derazne E., Tzur D., Gordon B., Tirosh A. (2013). White blood cells count and incidence of type 2 diabetes in young men. Diabetes Care.

[42] Vozarova B., Weyer C., Lindsay R.S., Pratley R.E., Bogardus C., Tataranni P.A. (2002). High white blood cell count is associated with a worsening of insulin sensitivity and predicts the development of type 2 diabetes. Diabetes.

[43] Lou M., Luo P., Tang R., Peng Y., Yu S., Huang W., He L. (2015). Relationship between neutrophil-lymphocyte ratio and insulin resistance in newly diagnosed type 2 diabetes mellitus patients. BMC Endocr. Disord..

[44] Shiny A., Bibin Y.S., Shanthirani C.S., Regin B.S., Anjana R.M., Balasubramanyam M., Jebarani S., Mohan V. (2014). Association of neutrophil-lymphocyte ratio with glucose intolerance: An indicator of systemic inflammation in patients with type 2 diabetes. Diabetes Technol. Ther..

[45] Sen N., Afsar B., Ozcan F., Buyukkaya E., Isleyen A., Akcay A.B., Yuzgecer H., Kurt M., Karakas M.F., Basar N. (2013). The neutrophil to lymphocyte ratio was associated with impaired myocardial perfusion and long term adverse outcome in patients with ST-elevated myocardial infarction undergoing primary coronary intervention. Atherosclerosis.

